# Frequency of silent brain infarction in transient global amnesia

**DOI:** 10.1007/s00415-021-10705-4

**Published:** 2021-07-17

**Authors:** Ramanan Ganeshan, Manja Betz, Jan F. Scheitz, Hebun Erdur, Heinrich J. Audebert, Jochen B. Fiebach, Kersten Villringer

**Affiliations:** 1 Department of Neurology, Charité, Universitätsmedizin Berlin, Berlin, Germany; 2 Center for Stroke Research Berlin, Charité, Universitätsmedizin Berlin, Berlin, Germany; 3 Berlin Institute of Health (BIH), Berlin, Germany; 4 DZHK (German Center for Cardiovascular Research), Partner Site Berlin, Berlin, Germany

**Keywords:** Transient global amnesia, MRI, Autonomic nervous system, Brain–heart syndrome

## Abstract

**Background and purpose:**

To determine the frequency and distribution pattern of acute DWI lesions outside the hippocampus in patients clinically presenting with Transient Global Amnesia (TGA).

**Methods:**

Consecutive patients clinically presenting with TGA between January 2010 and January 2017 admitted to our hospital were retrospectively evaluated. All patients fulfilled diagnostic criteria of TGA. We analyzed imaging and clinical data of all patients undergoing MRI with high-resolution diffusion-weighted imaging within 72 h from symptom onset.

**Results:**

A total of 126 cases were included into the study. Fifty-three percent (*n* = 71/126) presented with one or more acute lesions in hippocampal CA1-area. Additional acute DWI lesions in other cortical regions were found in 11% (*n* = 14/126). All patients with DWI lesions outside the hippocampus presented with neurological symptoms typical for TGA (without additional symptoms.)

**Conclusions:**

In a relevant proportion of clinical TGA patients, MRI reveals acute ischemic cerebral lesions. Therefore, cerebral MRI should be performed in patients with TGA to identify a possible cardiac involvement and to detect stroke chameleons.

## Introduction

Transient Global Amnesia (TGA) is a neurological disorder with sudden onset of anterograde amnesia which resolves by definition within 24 h [1]. Pathophysiological mechanisms underlying TGA are still not completely understood, but it is not considered to be attributable to ischemic stroke. However, ischemic stroke may cause acute transient amnesia too, and the differentiation from TGA may be difficult based on clinical presentation alone [2–4]. Magnetic resonance imaging (MRI) with high-resolution diffusion-weighted imaging (DWI) can provide relevant information in the diagnostic workflow [3]. Punctuate uni- or bilateral lesions in the hippocampal CA1-area on DWI are typical in TGA [5, 6], whereas lesion patterns may differ in patients with ischemic stroke, clinically presenting with amnesia [3]. Therefore, the aim of our study was to determine the frequency of ischemic lesions outside CA1 on high-resolution DWI in patients clinically presenting with TGA.

## Methods

### Study population

Consecutive patients clinically presenting with TGA between January 2010 and January 2017 admitted to our hospital were retrospectively evaluated. All patients were identified by searching our hospital digital patient records (SAP Clinical Workstation, SAP, Germany) for in-patients with the diagnosis TGA according to the International Classification of Diseases Tenth Revision. To reduce selection bias, we screened for TGA as suspected diagnosis in the emergency department and not for TGA as final diagnosis. MRI was normally performed the next morning after admission. Therefore, in patients with additional brain infarcts, the suspected diagnosis changed from TGA to final diagnosis stroke*.* Every patient was examined by a neurologist in the emergency department. TGA was defined according to established diagnostic criteria [7, 8]: witnessed attack, anterograde amnesia, no loss of consciousness or personal identity, cognitive impairment limited to amnesia; no other focal neurological symptoms during the attack or afterwards; no epileptic features or active epilepsy, no recent head injury, and resolution of symptoms within 24 h. Cerebral MRI was performed in all patients within 72 h from symptom onset. Patients with clinical TGA and uni- or bilateral isolated punctuate DWI lesion in the hippocampus were diagnosed as TGA. Patients with clinical TGA and additional acute ischemic lesions on DWI were diagnosed as ischemic stroke. Patients’ demographic and clinical data obtained included age, sex, time of symptom onset and cardiovascular risk factors. In accordance with the Berlin State legislation, no separate ethics committee approval was required for this retrospective analysis.

### Image acquisition and analysis

**Fig. 1:**
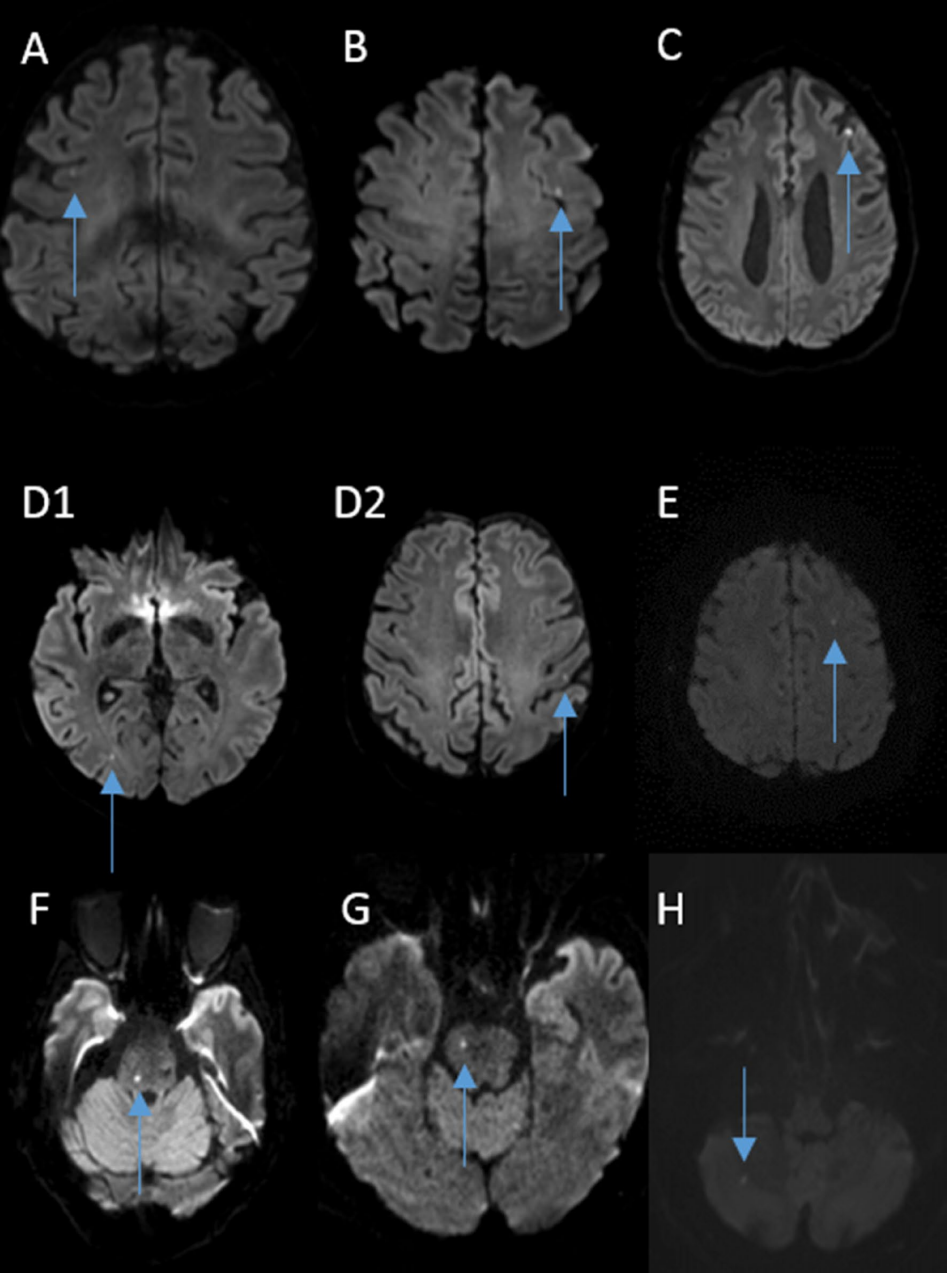
**A**–**H** Eight patients with acute ischemic DWI lesions in addition to the hippocampal lesion. **A**–**E** Five patients with ischemic lesions in the anterior circulation. **D1**–**D2** Same patient with two different ischemic lesions. **F**–**H** Three patients with additional lesions in the posterior circulation

All MRI examinations were performed on a 3 T MR scanner (Magnetom Trio; Siemens AG, Germany, 32-channel head coil). The MRI standard stroke protocol included high-resolution DWI, T2*-weighted imaging, MR-angiography and fluid attenuated inversion recovery (FLAIR). The sequence parameters for high-resolution DWI were: slice thickness 2.5 mm, repetition time TR 8900 ms, echo time TE 93 ms, slice gap 0, *b* values were 0 and 1000 mm*2/s. Besides the expected lesions in hippocampal CA1-area, DWI was screened for possible lesions (Fig. 1) at other locations outside the hippocampal level.

### Statistical methods

We calculated the frequency of patients with additional ischemic lesions on DWI in all patients clinically presenting with TGA over the entire observation period. For group comparisons between patients with and without additional ischemic lesions we used Mann–Whitney *U* test for continuous and *χ*^2^ test for categorical variables. In all analysis, a *P* value of < 0.05 (two-tailed) was used as a threshold of statistical significance. For data analysis, we used the Statistical Package for Social Sciences (SPSS Version 23, IBM, USA).

## Results

In total, we identified 126 patients with clinically diagnosed TGA and 3 T MRI within 72 h of symptom onset between January 2010 and January 2017, with a mean age of 66 (± 10) years; 66 (52%) were female. In addition to the 126 patients, we identified and excluded 78 patients with clinically diagnosed TGA in whom 3 T MRI was not performed for different reasons (contraindication for MRI, discharge before MRI could be performed). Table 1 shows characteristics of all patients with clinical TGA in whom 3 T MRI was performed and compares demographic and clinical characteristics of TGA patients with and without additional ischemic lesions on DWI. In 56% (71/126) of all patients, one or more acute punctuate DWI lesion were found in hippocampal CA1-areas. Unilateral hippocampal lesion occurred in 41% (52/126) and bilateral hippocampal lesions in 15% (19/126) of patients. In 11% (14/126), patients had additional acute DWI lesions (with corresponding ADC hypointensity) in brain areas outside the hippocampus. Cortical lesions in the anterior circulation occurred in eight patients, in the posterior circulation in one patients, in two patient subcortical lesions occurred in the posterior circulation. Three patients had cortical DWI lesions both in the anterior and posterior circulation. The frequency of coronary heart disease was significantly higher in patients with additional DWI lesions (25% vs. 6.8%, *p* = 0.034) while arterial hypertension and female sex were non-significantly more often observed in these patients. Age, frequency of previous stroke and other cardiovascular risk factors including atrial fibrillation did not differ between both the groups.

**Table 1:** Characteristics of all patients and patient with/without additional lesions on DWI

	Additional lesions *n* = 14 (11.1%)	No additional lesions *n* = 112 (88.9%)	*p*	Total *n* = 126 (100%)
Age in years, mean (SD)	69.14 (8.995)	66.05 (10.435)	0.292	66.4 (10.298)
Sex, female, *n* (%)	10 (71.4%)	56 (50%)	0.130	66 (52.4%)
Precipitating events				
Emotional trigger, *n* (%)	4 (36.4%)	19 (19.2%)	0.399	23 (20.9%)
Exhausting physical activity, *n* (%)	2 (20%)	27 (26.7%)	0.644	29 (26.1%)
Comorbidities				
Arterial hypertension, *n* (%)	11 (84.6%)	70 (62.5%)	0.114	81 (64.8%)
Coronary heart disease, *n* (%)	3 (25%)	7 (6.8%)	**0.034**	10 (8.7%)
Diabetes mellitus, *n* (%)	1 (7.1%)	9 (8.7%)	0.849	10 (8.5%)
Atrial fibrillation, *n* (%)	0 (0%)	10 (10%)	0.251	10 (8.9%)
Previous stroke, *n* (%)	1 (8.3%)	6 (5.8%)	0.724	7 (6.0%)
Hyperlipidemia, *n* (%)	5 (35.7%)	25 (24.3%)	0.358	30 (25.6%)
Number of cardiovascular risk factors, median (IQR)	1 (0–2)	1 (0.25–2)	0.483	1 (0–2)
Recurrent TGA, *n* (%)	1 (8.3%)	12 (11.2%)	0.762	13 (10.9%)
Clinical findings				
Systolic blood pressure > 140 mmHg on admission, *n* (%)	11 (84.6%)	84 (85.7%)	0.916	85 (85.6%)
Mean Systolic blood pressure on admission in mmHg (SD)	164.23 (31.776)	165.31 (27.118)	0.896	164.36 (27.546)
MRI findings DWI/T2				
Hippocampal lesion, *n* (%)	14 (100%)	57 (50.9%)	**< 0.001**	71 (56.3%)
Wahlund Score^12^, median (IQR)	4 (2.75–8)	4 (2–6)	0.337	4 (2–6)
Onset to imaging, < 24 h, *n* (%)	8 (57%)	67 (60%)	0.847	75 (59.5%)

Bold Values are statistically significant

*SD* standard deviation, *IQR* interquartile range, *DWI* diffusion-weighted imaging

## Discussion

In this 3 T MRI-study encompassing a large number of patients presenting with acute amnesia clinically thought to be TGA, about 10% of patients had acute lesions on high-resolution DWI outside the hippocampal level in addition to the expected and proven punctuate hippocampal lesions in the CA1-area. Acute and transient amnesia in TGA is suspected to be caused by a transient disturbance in the hippocampal functional memory network. Hence, in many patients clinically presenting with TGA, a punctuate DWI lesion can be detected in the hippocampal area [1]. But recent evidence showed that transient amnesia can be provoked by ischemic lesions affecting the hippocampal circuit, labeled as ischemic amnesia [3, 5]. In one of these studies, 1.2% of patients with acute amnesia were later shown to be due to cerebral ischemia, but the true rate of ischemic amnesia could not be estimated, since only < 25% of clinical TGA patients underwent MRI (1.5 and 3 T) and information regarding DWI image resolution was not available. Second, only two patients with ischemic amnesia were considered certain TGAs before imaging because they fulfilled all clinical TGA criteria. In our stroke center, we regularly perform 3-Tesla MR-imaging in patients clinically presenting with TGA to identify stroke chameleon. More importantly, we use a high-resolution DWI with a slice thickness of 2.5 mm and no slice gap enabling the detection of small lesions otherwise undetected at lower image resolution. This might explain the high rate of patients with additional DWI lesions outside the hippocampal level of more than 10% in our cohort. Additional acute lesions predominantly occurred in the anterior circulation or in multiple vascular territories, the latter suggesting cardiac (or aortic arch) involvement. This might be substantial, as the hippocampus is regarded as the “forgotten” border zone of brain ischemia [2]. In patients with additional acute lesions, coronary heart disease and arterial hypertension were more frequent, increasing the risk of acute cardiac dysfunction. Taking all together, intense emotional or physical stress as a frequent precipitating event with excessive sympathetic activation might elicit cardiac diastolic and/or systolic output dysfunction resulting in brain hypoperfusion compromising susceptible brain structures such as the hippocampus. This possible pathophysiology of TGA and ischemic amnesia is supported by a recently published report presenting evidence that the likelihood of myocardial injury in patients with TGA was twofold higher compared to patients with transient ischemic attack [9]. In another recent study nearly 9% of TGA patients had elevated levels of high sensitive troponine T, suggesting myocardial injury [10]. Furthermore, the likelihood of a history of coronary heart disease was significantly greater in these patients. In addition, in two patients, a Takotsubo syndrome (TTS) was diagnosed. TTS is an acute but transient left ventricular cardiac dysfunction with a preceding emotional or physical stressful event, analogical to TGA patients. This may indicate a pathophysiological correlation between TGA and TTS, supporting the hypothesis that TGA may be associated with acute cardiac dysfunction leading the further brain injury. Hence, cerebral MRI with high-resolution DWI and cardiovascular workup provides important information in patients clinically presenting with TGA to detect additional acute lesions possibly related to cardiac dysfunction or myocardial injury.

Limitations of our study include the retrospective and the single-center design increasing the risk of selection bias. Not all patients with clinical TGA underwent 3 T MRI because of contraindication for performing MRI or because they were discharged before 3 T MRI was available. Furthermore, cardiovascular and neuropsychological testing was not systematically performed. Strengths are the homogeneity of our cohort with systematically acquired high-resolution DWI in most patients clinically presenting with TGA over a long study time period.

## Conclusions

Acute ischemic lesions in patients presenting clinically as TGA are obviously more frequent than previously thought. Therefore, cerebral MRI should be performed in patients with clinical TGA to detect ischemic lesions possibly related to cardiac dysfunction or myocardial injury.

## Data Availability

Anonymized data and methods will be shared by request from any qualified investigator for analysis at the Center for Stroke Research Berlin. Data will be shared to noncommercial and academic purposes only. The corresponding author will keep a copy of the final dataset for at least 10 years.
